# Amentoflavone and cancer: mechanistic insights and translational perspectives

**DOI:** 10.3389/fonc.2026.1813172

**Published:** 2026-07-30

**Authors:** Hiroki Hashida, Atsushi Kobayashi, Masatoshi Akagami, Tomohiro Okamoto, Hirokazu Tanaka

**Affiliations:** Department of Gastrointestinal Surgery, Hanwa Memorial Hospital, Osaka, Japan

**Keywords:** amentoflavone, angiogenesis, apoptosis, biflavonoid, EMT, formulation, NF-κB, pharmacokinetics

## Abstract

Amentoflavone (AF) is a naturally occurring biflavonoid isolated from multiple medicinal plants and widely studied for anti-inflammatory and antioxidant activities. In oncology, AF has emerged as a pleiotropic small molecule that modulates interconnected signaling axes rather than a single node. Preclinical studies indicate that AF attenuates PI3K/Akt/mTOR signaling and NF-κB–dependent survival programs, disrupts Wnt/β-catenin–linked epithelial–mesenchymal transition (EMT), and modulates MAPK cascades to promote apoptosis and cell-cycle arrest. AF also interferes with pro-angiogenic signaling and VEGF-driven neovascularization. Across diverse cancer models, these activities translate into reduced proliferation, enhanced apoptosis, suppression of invasion/EMT, and inhibition of tumor-associated angiogenesis. However, translation remains limited by poor aqueous solubility, low oral exposure, and incomplete exposure–response and safety characterization. Recent drug delivery approaches (polymeric micelles, mixed nanomicelles, DSPE-PEG micelles) and rational combination strategies provide practical routes to improve systemic exposure and revisit AF in combination regimens. This review integrates mechanistic and tumor-specific evidence with a clear distinction between direct target binding and downstream pathway readouts, quantitative *in vitro*/*in vivo* endpoints, an expanded reference list using verifiable sources, and priorities for pharmacokinetic optimization, toxicity evaluation, and biomarker-guided early-phase development.

## 1 Introduction

Natural products remain an important source of anticancer agents and lead structures, and multi-target small molecules are increasingly attractive for suppressing network-level survival and invasive programs (1, 2).

Amentoflavone (AF; **Supplementary Figure 1**) is a plant-derived biflavonoid present in several medicinal species (e.g., Selaginella and Ginkgo). Comprehensive reviews describe broad pharmacological activities including anti-inflammatory, antiviral, and antitumor effects (3–5).

In oncology models, AF has been reported to influence multiple hallmarks of cancer by modulating signaling hubs frequently co-activated in solid tumors—PI3K/Akt/mTOR, NF-κB, Wnt/β-catenin, MAPK, and pro-angiogenic VEGF signaling—thereby affecting apoptosis, EMT, invasion, and angiogenesis (3, 5, 6).

A key barrier to clinical translation is pharmacokinetic: AF has poor aqueous solubility and low oral exposure with extensive metabolism, and oral absorption risk assessments and rat pharmacokinetic studies support these limitations (3, 7–9).

Accordingly, formulation strategies (e.g., oral polymeric micelles, mixed nanomicelles, DSPE-PEG micelles) have been developed to improve dispersibility and bioavailability and to enable measurable systemic exposure (10–12).

Here, we synthesize current evidence on AF by integrating mechanistic studies, tumor-specific findings, quantitative *in vitro*/*in vivo* endpoints, pharmacokinetic and formulation data, safety considerations, and translational barriers, with particular attention to distinguishing direct target-binding evidence from downstream pathway readouts.

## 2 Molecular mechanisms of anticancer action

**Figure 1:**
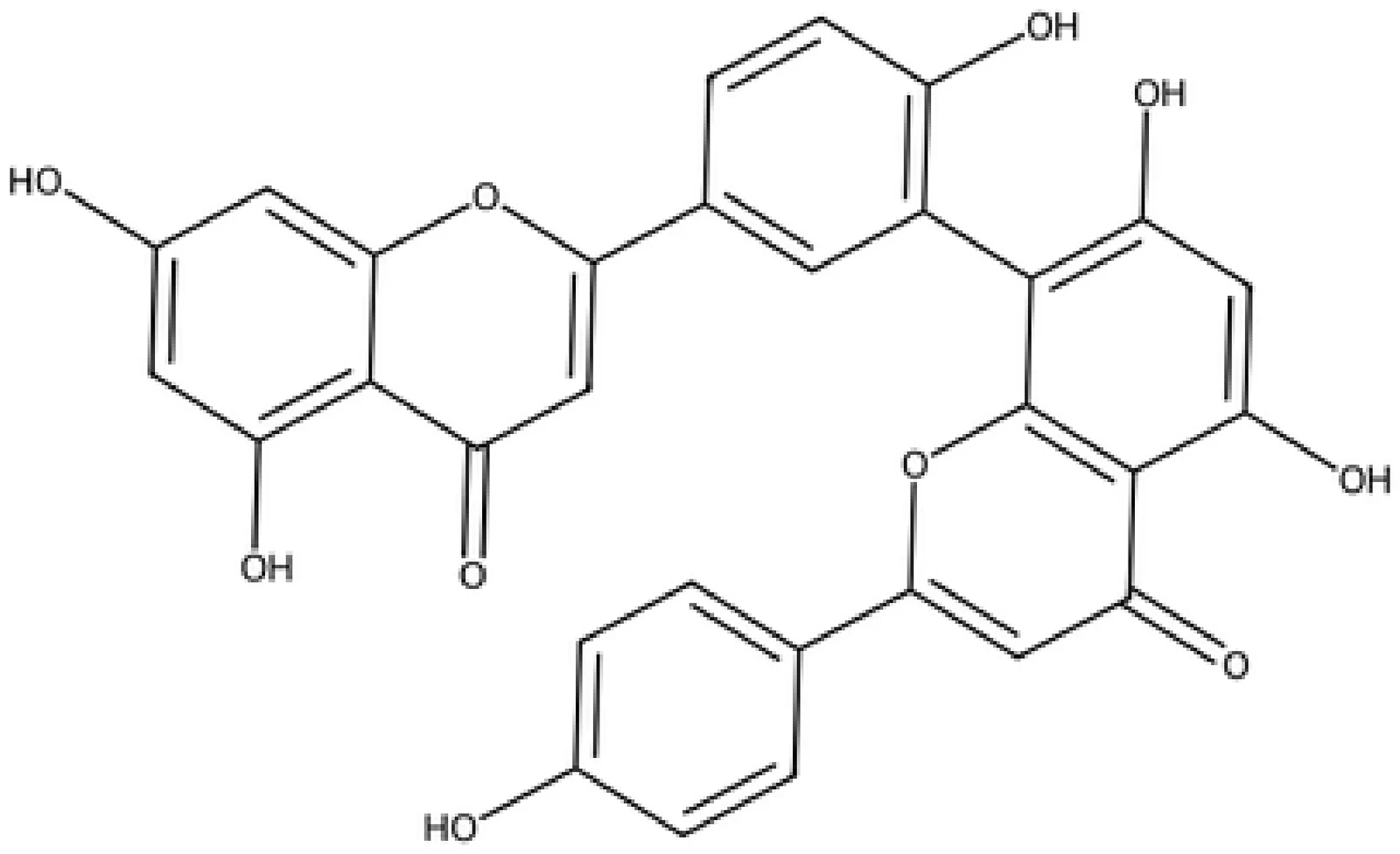
Updated schematic of AF-modulated signaling hubs with representative molecular nodes. AF influences PI3K/Akt/mTOR, NF-κB, MAPK, Wnt/β-catenin/EMT programs, and pro-angiogenic VEGF signaling, leading to apoptosis induction, EMT suppression, and inhibition of neovascularization.

AF influences multiple signaling modules (**Figure 1**; **Table 1**) and may function as a systems-level modulator (3, 5).

**Table 1:** Key signaling modules modulated by amentoflavone (selected examples).

Pathway/module	Representative readouts	Functional outcome (reported)
PI3K/Akt/mTOR	↓ p-Akt, ↓ mTOR signaling (mostly downstream readouts)	Reduced proliferation; apoptosis sensitization
NF-κB	↓ NF-κB activation, ↓ COX-2/cyclin D1/survival genes	Anti-inflammatory; pro-apoptotic; anti-invasive
Wnt/β-catenin/EMT	miR-16-5p↑; HMGA2↓; β-catenin↓; EMT marker shift	Reduced migration/invasion; EMT suppression
Angiogenesis (VEGF family)	↓ VEGF-driven neovascularization (functional assays)	Reduced neovascularization; tumor growth inhibition
MAPK (ERK/JNK/p38)	ERK phosphorylation↓ in some models; stress MAPK modulation	Cell-cycle arrest; apoptosis

↓, decreased/reduced or inhibited; ­↑, increased/upregulated.

### 2.1 Evidence hierarchy: direct binding vs downstream signaling

Because “targets” are often inferred from downstream signaling changes, we classify evidence into: (i) direct binding/biochemical interaction (e.g., enzymatic inhibition, ligand–receptor interference), (ii) cellular target engagement assays, and (iii) downstream pathway readouts (e.g., phosphorylation, transcriptional reporters). In many AF studies, pathway modulation is supported mainly by category (iii), whereas a smaller subset provides category (i) evidence (e.g., inhibition of VEGF-driven neovascularization through interference with pro-angiogenic VEGF family activity) (6, 13).

When AF is reported to “inhibit NF-κB,” for example, this may refer to reduced NF-κB DNA-binding/translocation or reduced NF-κB-dependent gene expression; direct binding claims are typically based on in silico docking and require independent biochemical validation (14, 15).

### 2.2 Regulation of PI3K/Akt/mTOR

The PI3K/Akt/mTOR axis regulates growth, metabolism, and survival and is frequently activated in solid tumors. Multiple studies summarized in recent reviews report that AF decreases Akt phosphorylation and downstream mTOR signaling, aligning with reduced proliferation and increased apoptotic signaling (3, 5).

Because PI3K/Akt cross-talks with NF-κB and MAPK, coordinated attenuation may suppress adaptive resistance responses to chemotherapy or targeted therapy (5, 16).

### 2.3 Inhibition of NF-κB signaling

NF-κB orchestrates inflammatory and anti-apoptotic transcriptional programs that support progression and therapy resistance. In TNFα-activated A549 cells, AF inhibited COX-2 induction with concomitant inhibition of NF-κB signaling and effects on IκBα degradation/translocation, supporting NF-κB pathway suppression at the signaling level (14).

In a recent *in vitro* A549 study, AF reduced viability (notably at 60 μM) and induced apoptosis while suppressing NF-κB activity and NF-κB-associated survival genes (Bcl-XL, Bcl-2, survivin) and cyclin D1; the study also reported in silico docking to NF-κB p65/p50, which should be considered hypothesis-generating for direct binding (15).

### 2.4 Modulation of Wnt/β-catenin and EMT programs

Aberrant Wnt/β-catenin signaling supports EMT, stem-like states, and metastatic dissemination in gastrointestinal malignancies. The strongest CRC-specific mechanistic evidence links AF to an miR-16-5p/HMGA2/β-catenin axis, connecting AF exposure to reduced β-catenin signaling and EMT suppression (17).

Because EMT is a plastic spectrum rather than a binary switch, future studies should quantify EMT-related transcriptional signatures and functional endpoints (migration/invasion, circulating tumor cell surrogates, metastatic burden) under exposure-controlled dosing (16, 18). AF has also been reported to modulate matrix metalloproteinase programs in non-malignant cellular contexts, suggesting potential relevance to invasion biology (19).

### 2.5 Suppression of angiogenesis and hypoxia-adaptation pathways

Angiogenesis is a key hallmark of progression and metastasis. AF inhibited VEGF-A-induced and PlGF-1-induced neovascularization and reduced tumor growth and associated neovascularization *in vivo*, providing a rare example of functional anti-angiogenic evidence beyond signaling readouts (6).

Independent work also reported inhibition of tumor-specific angiogenesis by AF *in vivo* (13).

In breast cancer MCF-7 cells, AF induced anti-angiogenic and anti-metastatic effects through suppression of NF-κB activation, linking inflammatory transcriptional control to angiogenic outputs (20).

## 3 Tumor-specific evidence

**Figure 2:**
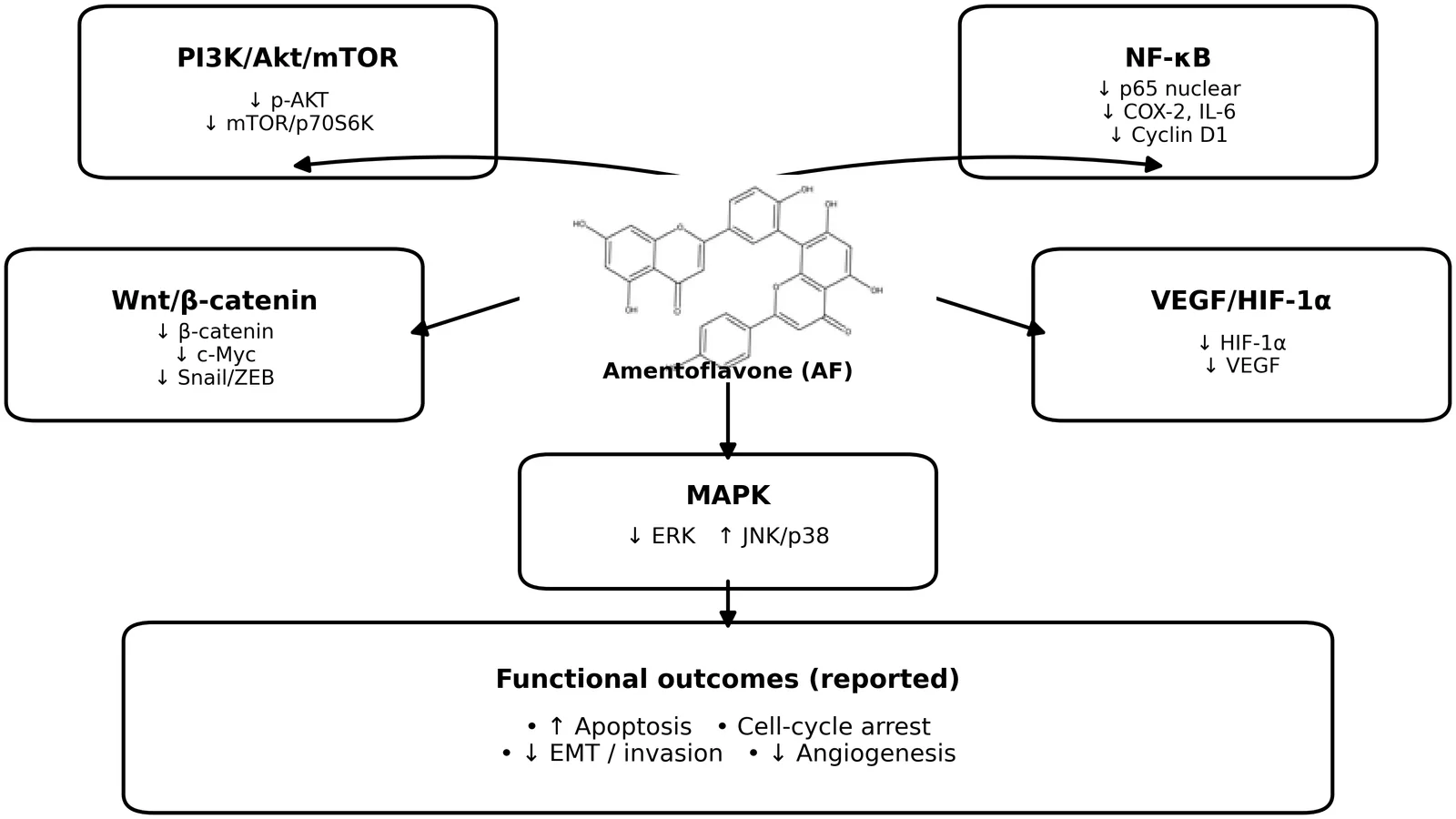
Tumor–pathway map of reported AF targets across representative malignancies. Legend is embedded within the figure (filled symbols: frequently reported modulation;open/gray symbols: limited or mixed evidence).

**Figure 3:**
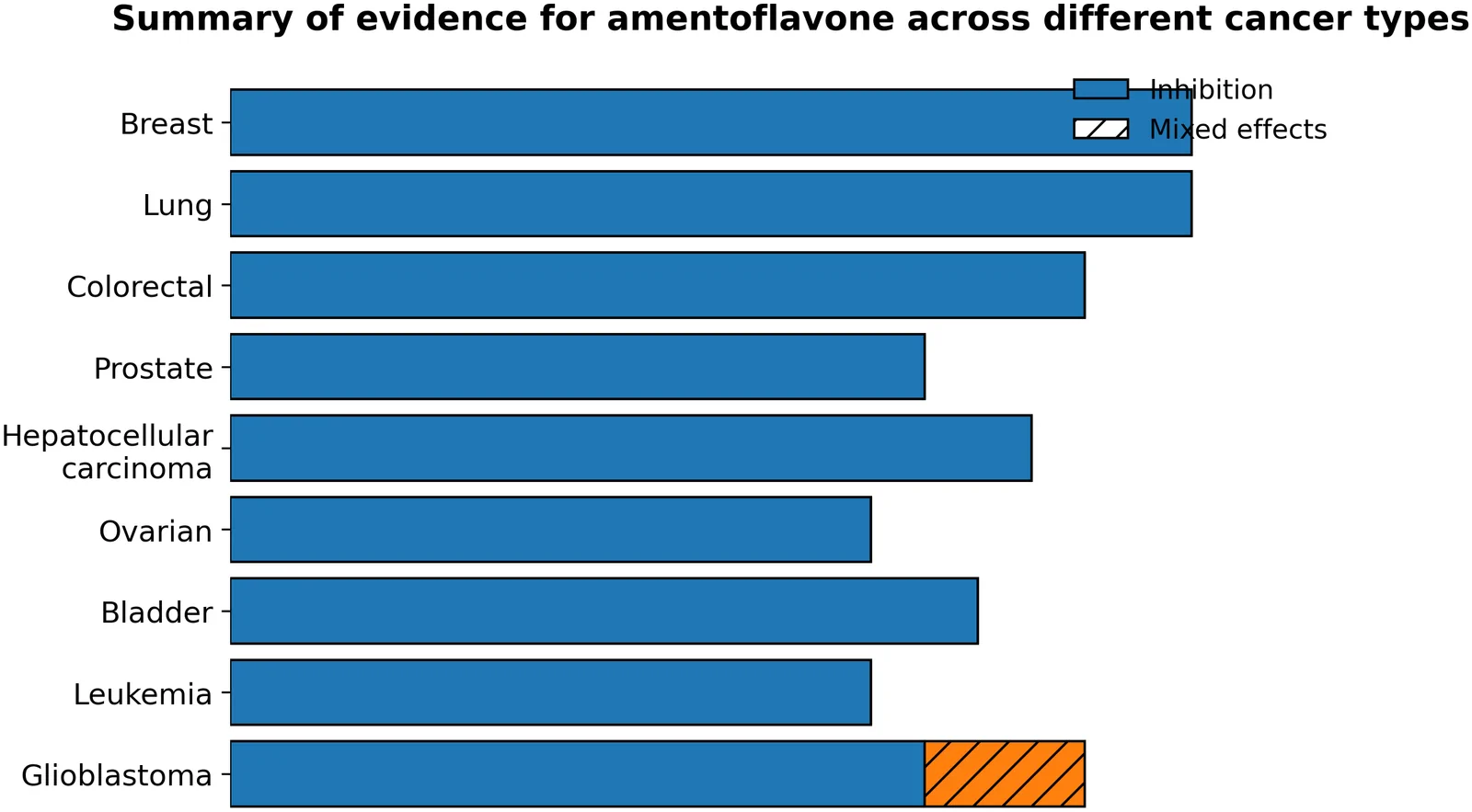
Summary of tumor-type evidence for AF across cancer types (qualitative).

Tumor-level evidence across cancer types is summarized schematically in **Figure 2**, and tumor–pathway mapping is provided in **Figure 3**. We focus on studies that report functional endpoints (apoptosis, invasion/EMT, angiogenesis) and, where available, quantitative exposure and dosing details.

### 3.1 Colorectal cancer

A CRC-focused study provides a mechanistic anchor for AF as an anti-metastatic agent: in CRC cells and patient-derived xenograft (PDX) models, AF suppressed EMT via the miR-16-5p/HMGA2/β-catenin pathway, linking AF exposure to inhibition of Wnt-driven invasive programs (17).

#### 3.1.1 EMT and metastatic programs

*In vitro*, AF (5 μM) reduced migration and invasion of HCT116 and SW480 cells and shifted EMT marker expression toward an epithelial phenotype (↑E-cadherin; ↓N-cadherin/vimentin) (17).

Mechanistically, AF increased miR-16-5p and downregulated HMGA2 with downstream inhibition of β-catenin signaling and EMT transcriptional outputs; reporter assays supported HMGA2 as an miR-16-5p target (17).

#### 3.1.2 *In vivo* activity and biomarker-oriented translation

In a CRC PDX model, daily oral AF (50 mg/kg) for 45 days inhibited tumor growth and reduced HMGA2/β-catenin pathway activity, supporting an exposure-linked anti-EMT and anti-tumor effect under a chronic dosing schedule (17).

The same work reported translational correlative data in 96 CRC specimens, where higher miR-16-5p and lower HMGA2 levels were associated with improved overall survival, suggesting a potential pharmacodynamic/biomarker axis for early-phase proof-of-mechanism designs (17).

#### 3.1.3 Combination strategies in CRC: what is known and what remains

While chemosensitization is frequently proposed for multi-target flavonoids, direct, reproducible CRC-specific synergy data for AF remain limited. We therefore treat combination strategies in CRC as a research priority rather than an established property.

Future work should evaluate AF combinations under exposure-controlled conditions in resistant CRC models (organoids/PDX-derived systems), guided by known resistance routes (e.g., EMT and Wnt-related programs) and general principles of flavonoid-mediated sensitization (16, 18). Verified combination evidence and priorities for future studies are summarized in **Table 2**.

**Table 2:** Combination strategy evidence (verified examples) and priorities.

Tumor type	Combination agent	Model (reported)	Observed outcome (reported)	Reference
HCC	Sorafenib	SK-Hep1R (*in vitro*)	AF promoted sorafenib-induced apoptosis in resistant cells.	(21)
HCC	Sorafenib	*In vivo* HCC model	Combination enhanced therapeutic efficacy and apoptosis vs sorafenib alone.	(22)
CRC	— (priority for future studies)	Organoid/PDX-derived models (recommended)	Need for rigorous synergy testing; focus on EMT/Wnt and NF-κB stress responses.	(16, 18)

### 3.2 Lung cancer

In lung cancer models, AF has been studied primarily as a modulator of inflammatory survival signaling. In TNFα-activated A549 cells, AF inhibited COX-2 induction and NF-κB signaling, supporting a mechanistic link between NF-κB suppression and downstream inflammatory gene control (14).

*In vitro* A549 work reported concentration-dependent cytotoxicity and apoptosis induction with NF-κB suppression; at 60 μM, cell viability was substantially reduced and apoptotic markers increased, accompanied by decreased expression of NF-κB-associated survival genes (15).

Given heterogeneity in exposure ranges across studies, quantitative endpoints for representative lung studies are summarized in **Table 3**.

**Table 3:** Quantitative endpoints reported in representative AF studies (selected).

Tumor type	Model	AF exposure/dose	Quantitative endpoint(s)	Mechanistic readout(s)	Ref
CRC	HCT116/SW480 (*in vitro*)	5 μM	↓ migration/invasion; EMT marker shift	miR-16-5p↑; HMGA2↓; β-catenin↓	(17)
CRC	CRC PDX (*in vivo*)	50 mg/kg p.o. daily ×45 days	Tumor growth inhibition (PDX)	HMGA2/β-catenin axis suppressed	(17)
Lung	A549 (*in vitro*)	60 μM (24 h) noted; 30–120 μM used	Viability reduction; apoptosis induction	NF-κB suppression; survival genes↓	(15)
Angiogenesis	CAM/tumor models (*in vivo*)	μM range in functional assays	↓ VEGF-A/PlGF-1 neovascularization; ↓ tumor neovascularization	Interference with pro-angiogenic VEGF family activity	(6)

↓, decreased/reduced or inhibited; ­↑, increased/upregulated.

### 3.3 Hepatocellular carcinoma

In hepatocellular carcinoma (HCC) SK-Hep1 models, AF reduced ERK phosphorylation, NF-κB activation, and invasion-associated phenotypes, consistent with suppression of progression-associated programs (23).

AF has also been evaluated as a sensitizer for sorafenib in HCC. In sorafenib-resistant SK-Hep1R cells, AF promoted sorafenib-induced apoptosis *in vitro* (21). *In vivo*, combination therapy enhanced anticancer efficacy of sorafenib by reducing anti-apoptotic potential and potentiating apoptosis (22).

### 3.4 Breast cancer

In MCF-7 breast cancer cells, AF induced cell-cycle arrest and mitochondria-dependent apoptosis (caspase-dependent and -independent components have been described) (24).

Separately, AF induced anti-angiogenic and anti-metastatic effects in MCF-7 cells through suppression of NF-κB activation, providing a mechanistic link to reduced angiogenic outputs (20).

Additionally, AF has been reported to induce apoptosis in human cervical cancer cells by suppressing HPV E7 expression and downstream COX-2/IL-32 activation, underscoring anti-inflammatory and pro-apoptotic coupling beyond breast models (25).

Claims of clinically meaningful radiosensitization remain insufficiently supported in the AF literature; therefore, we limit radiation-related statements to future research directions.

### 3.5 Other tumor models and limitations

AF activity has also been reported in other tumor models including osteosarcoma, where AF reduced metastatic potential through suppression of ERK and NF-κB activation (26).

In osteosarcoma, AF has also been evaluated as a sensitizer to sorafenib, with reported reinforcement of sorafenib efficacy accompanied by apoptosis induction and ERK/NF-κB inactivation (27).

Evidence in glioblastoma and other difficult-to-treat tumors remains comparatively limited, and key constraints include blood–brain barrier penetration and exposure–response characterization. Accordingly, we avoid broad mechanistic claims in these settings and emphasize the need for formulation-enabled exposure and clinically relevant *in vivo* models (3, 7–9).

## 4 Pharmacokinetics and formulation strategies

Poor aqueous solubility and extensive metabolism contribute to low oral exposure of AF and limit translation. Rat pharmacokinetic work and absorption risk evaluation support the need for exposure optimization and standardized PK reporting (3, 7–9).

Several oral micelle-based delivery approaches have reported improved bioavailability. In one polymeric micelle system, oral administration increased AF bioavailability by ~3.2-fold versus solution (10). DSPE-PEG2000 micelles reported ~3.79-fold enhanced oral bioavailability and improved *in vitro* antitumor efficacy (12). Mixed nanomicelles using TPGS/Soluplus have also been developed to improve solubility and systemic exposure (11). Cyclodextrin inclusion complexes represent another established strategy for improving the apparent solubility and delivery of poorly water-soluble compounds, although AF-specific pharmacokinetic evidence for this approach remains limited (28).

Beyond exposure, metabolite identification studies have begun to map AF biotransformation *in vivo* (plasma, bile, urine, feces) and *in vitro* (liver and intestinal microsomes), which is essential for interpreting efficacy, safety, and potential drug–drug interactions (29).

We emphasize that improved dispersibility does not guarantee tumor delivery; future work should pair formulation PK (Cmax/AUC) with pharmacodynamic biomarkers (e.g., miR-16-5p/HMGA2 axis in CRC) and systematic toxicity evaluation (11, 12, 17).

### 4.1 Safety, toxicity, and drug–drug interaction considerations

**Figure 4:**
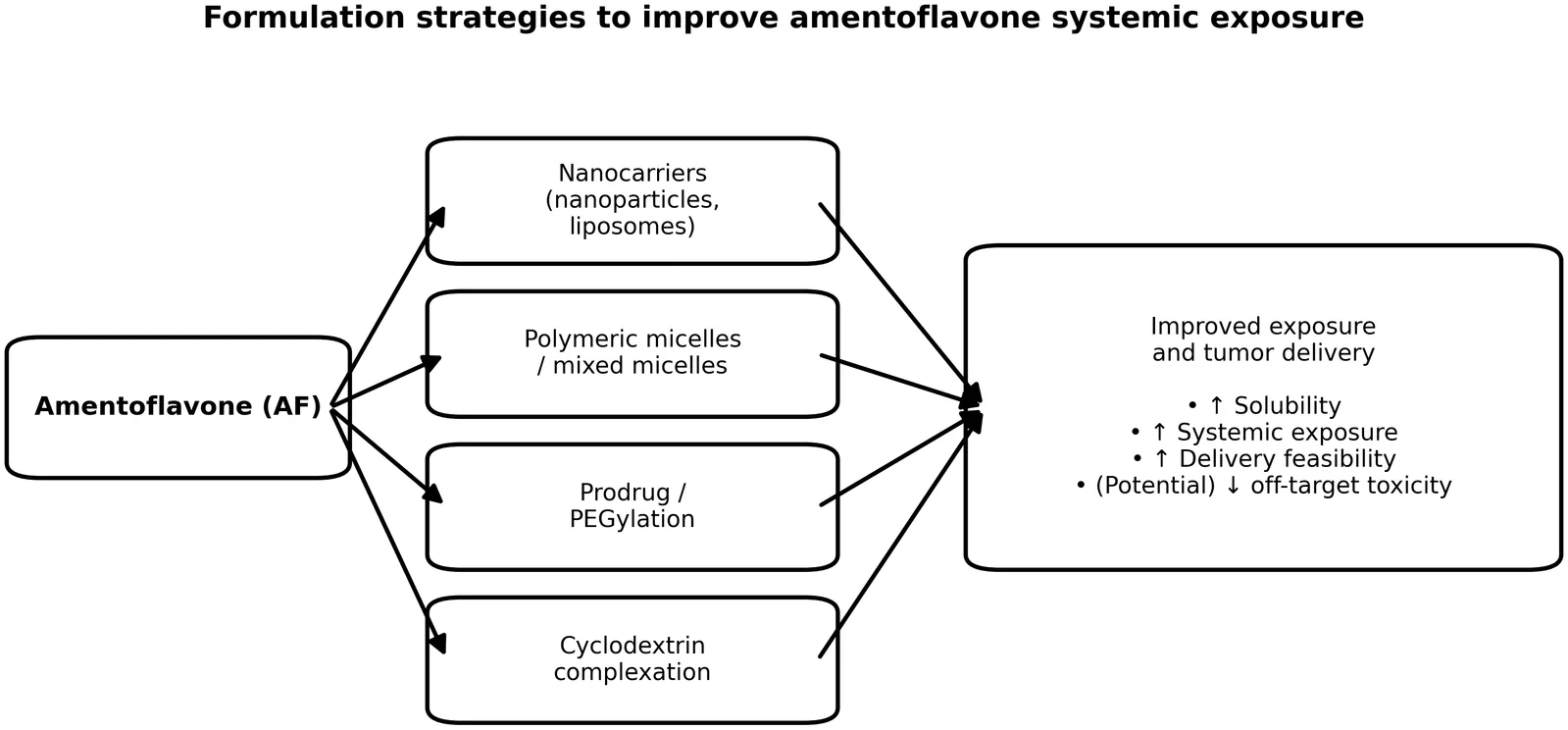
Overview of formulation strategies to improve AF systemic exposure (micelles, mixed nanomicelles, DSPE-PEG micelles) and the need to link PK to PD biomarkers.

Efficacy claims for AF should be interpreted alongside a critical assessment of safety, toxicity, and drug–drug interaction risks. Across the AF oncology literature, *in vivo* studies often report limited gross toxicity (e.g., stable body weight) at efficacy-relevant dosing schedules, but detailed toxicology (clinical chemistry, histopathology, chronic dosing, and metabolite-linked liabilities) is frequently absent. Formulation-enabled exposure increases (**Table 4**; **Figure 4**) therefore require parallel safety evaluation of both AF and excipients (e.g., TPGS/Soluplus or DSPE-PEG systems), with standardized reporting of adverse events, organ toxicity, and exposure–response relationships. At minimum, future studies should include dose-escalation with predefined stopping rules, hematology/chemistry panels, and histopathology of liver, kidney, and GI tract, together with plasma and tissue exposure to define margins relative to pharmacodynamic activity (3, 7, 10–12).

**Table 4:** Selected formulation strategies and reported pharmacokinetic improvements.

Formulation approach	Key note	Evidence
Polymeric micelles (P(NVP-MGAM)/AF)	Oral micelles increased bioavailability ~3.2× vs solution (KKAy mice study context).	(10)
Mixed nanomicelles (TPGS/Soluplus)	Developed to improve solubility/bioavailability and enable measurable systemic exposure and metabolite profiling.	(11)
DSPE-PEG2000 micelles	Reported improved oral bioavailability (~3.79×) and improved *in vitro* antitumor efficacy.	(12)

An additional translational risk is drug–drug interaction (DDI). Amentoflavone has been reported as a potent inhibitor of major human CYP enzymes, including CYP3A4 and CYP2C9, with submicromolar IC50 values *in vitro*, raising a plausible risk for clinically meaningful exposure increases of co-administered drugs (30). More recent human liver microsome studies in a Selaginella extract context similarly identify amentoflavone as a major contributor to broad CYP inhibition and time-dependent inhibition for selected isoforms, supporting the need to assess DDI risk early if AF is advanced toward clinical testing (31). Accordingly, any early-phase development should prospectively evaluate CYP/UGT inhibition, transporter interactions, and formulation-dependent changes in exposure before combination regimens are pursued.

## 5 Microenvironmental and immunometabolic considerations

AF has well-described anti-inflammatory activity largely through NF-κB suppression and downstream inflammatory mediators, which suggests a plausible capacity to modulate pro-tumor inflammation (3, 14, 32).

However, direct tumor microenvironment (TME) studies quantifying immune composition, spatial biology, and functional antitumor immunity remain sparse for AF. We therefore treat immunometabolic claims cautiously and recommend that future work explicitly measures immune and stromal endpoints in immunocompetent models alongside exposure (3, 5).

## 6 Translational perspectives and future outlook

To progress AF toward clinical evaluation, three prerequisites are repeatedly apparent: (i) exposure optimization (formulation and PK), (ii) safety/toxicity characterization (including chronic dosing and metabolite profiling), and (iii) biomarker-guided proof-of-mechanism endpoints (3, 7–12, 17).

Because AF modulates multiple signaling hubs, it may be best positioned as an adjunct to standard therapy rather than a standalone cytotoxic agent. Combination strategies should be evaluated with rigorous synergy methods and with attention to drug–drug interaction potential, including CYP inhibition reported for amentoflavone (3, 16).

### 6.1 Clinical development landscape and barriers

An explicit clinical development landscape is important for contextualizing the translational status of AF. We searched major public trial registries (ClinicalTrials.gov and the WHO ICTRP) for interventional oncology studies listing amentoflavone as an intervention (accessed 9 June 2026) and did not identify registered anticancer trials (33, 34). This absence likely reflects combined scientific and practical barriers: low oral exposure with uncertain human pharmacokinetics, limited formal toxicology packages, and the challenges of developing natural products where intellectual property, quality control, and funding pathways can be less straightforward than for novel synthetic entities (1, 35).

Regulatory pathways for botanical products emphasize consistent chemistry/manufacturing controls and early alignment on development plans (35, 36). The FDA botanical drug development guidance highlights CMC standardization and staged nonclinical-to-clinical transitions, which can be instructive for AF development even if AF is pursued as a purified small molecule rather than a complex botanical mixture (35). From a pragmatic standpoint, an AF translational program should prioritize (i) formulation-enabled exposure with validated bioanalytical assays, (ii) standardized safety and DDI evaluation (Section 4.1), and (iii) biomarker-guided endpoints (e.g., EMT signatures such as miR-16-5p/HMGA2 in CRC) to justify early-phase studies.

## 7 Conclusions

AF is a multi-target biflavonoid with preclinical evidence supporting apoptosis induction, EMT/invasion suppression, and anti-angiogenic activity across tumor models. The most coherent CRC mechanistic chain links AF to miR-16-5p/HMGA2/β-catenin signaling and EMT suppression, supported by both *in vitro* and PDX evidence (17).

Translation will require improved exposure, standardized quantitative reporting, and systematic toxicity evaluation. Formulation approaches that increase bioavailability provide a practical starting point, but must be coupled to pharmacodynamic biomarkers and safety assessment to enable early-phase clinical testing (3, 7–12).

## Supplementary material

Supplementary Figure 1 Chemical structure of amentoflavone.
