# Correction: Amentoflavone and cancer: mechanistic insights and translational perspectives

**DOI:** 10.3389/fonc.2026.1955891

**Published:** 2026-08-19

**Authors:** Hiroki Hashida, Atsushi Kobayashi, Masatoshi Akagami, Tomohiro Okamoto, Hirokazu Tanaka

**Affiliations:** Department of Gastrointestinal Surgery, Hanwa Memorial Hospital, Osaka, Japan

**Keywords:** amentoflavone, angiogenesis, apoptosis, biflavonoid, EMT, formulation, NF-κB, pharmacokinetics

In the published article, the captions of **Figure 2** and **Figure 3** were inadvertently interchanged. The figure images and the scientific content are correct; only the captions require correction.

The corrected caption for **Figure 2** is:

“Tumor–pathway map of reported AF targets across representative malignancies. Legend is embedded within the figure (filled symbols: frequently reported modulation; open/gray symbols: limited or mixed evidence).”

The corrected caption for **Figure 3** is:

“Summary of tumor-type evidence for AF across cancer types (qualitative).”

This correction does not change the scientific conclusions of the article in any way.

The original version of this article has been updated.

